# Review of hTERT-Immortalized Cells: How to Assess Immortality and Confirm Identity

**DOI:** 10.3390/ijms252313054

**Published:** 2024-12-04

**Authors:** Maria Shitova, Elena Alpeeva, Ekaterina Vorotelyak

**Affiliations:** Laboratory of Cell Biology, N.K. Koltzov Institute of Developmental Biology of Russian Academy of Sciences, Vavilov Street 26, 119334 Moscow, Russia; alpeeva_l@mail.ru (E.A.); vorotelyak@yandex.ru (E.V.)

**Keywords:** immortalization, immortalized cells, telomerase, telomere length, hTERT, replicative senescence, karyotype, TRAP

## Abstract

Cell immortalization has an important role in scientific research, as well as increasing significance in the context of cell therapy and biotechnology. Over the years, many immortalized cell lines have been produced using human telomerase reverse transcriptase (hTERT) alone or in a combination with viral oncogenes. Different hTERT-immortalized cells are commercially available, and numerous papers about obtaining immortalized cell lines have also been published. However, no specific list of characteristics that need to be checked to confirm successful immortalization exists. Most researchers evaluate only a few parameters, while different articles contain various opinions on the assessment of these characteristics. Results also vary significantly between different cell types, which have their own traits depending on their origin and functions. In the current paper, we raise these questions and discuss controversial issues concerning currently available testing methods for immortalization evaluation and the value and the limitations of the approaches. In addition, we propose a protocol for evaluation of hTERT immortalization success consisting of the following important steps: the assessment of the proliferation rate and dividing capacity, cell morphology, phenotype, karyotype stability, telomerase activity, the expression of cell-specific markers, and tumorigenicity. To our opinion, the hTERT expression level, telomere length, and senescence-associated β-galactosidase staining are controversial with regard to the implemented methods, so these parameters may be optional. For all the evaluation steps, we recommend to pay attention to the necessity of comparing the traits of the obtained immortalized and parent cells.

## 1. Introduction

Different cell lines and primary cultures are widely used in the laboratory and in industrial and clinical practices. Normal diploid cells have a limited lifespan in vitro, but immortal cells (such as cancer and immortalized) can divide infinitely, which facilitates their use in scientific research. Nevertheless, cancer cells usually possess aberrant karyotype and altered traits, while cultured primary cells retain a morphology and physiology similar to those of the cells in vivo. Immortalized cells stand between these two types, having properties more like primary diploid cells but possessing unlimited dividing capacity (we suggest that it is more accurate to define as “extended dividing capacity”), which is quite important for in vitro experiments. Along with the development of cell biology aimed at clinical implementation, approaches for the immortalization of cells became more in demand. Nevertheless, there is no consistent description of the characteristics for the confirmation of cell immortalization, while different scientific papers contain various opinions on the assessment of this parameter.

Telomeres are repeating DNA sequences (TTAGGG), which stabilize the ends of chromosomes acting like caps. During each cell division, 50–200 base pairs of DNA are lost at the ends of chromosome telomeres [1]. Telomere critical shortening is thought to trigger the cell DNA damage response, leading to irreversible cell cycle arrest and replicative senescence due to incomplete lagging DNA strand synthesis and end-processing events [2,3,4]. Telomerase is a reverse transcriptase (abbreviated to TERT, or hTERT in humans) comprising an RNA component (i.e., hTERC or hTR) and a catalytic component (i.e., hTERT), which adds DNA telomeric repeats at the 3′ ends of chromosomes [5]. In cells with active telomerase, the chromosome length is maintained, and the cells can potentially divide without senescence [1]. The RNA component of telomerase is expressed in most cells of the adult organism [6], while the expression of the catalytic component of the enzyme takes place in germ cells, the proliferative stem cells of renewing tissues, and cancer cells. Consequently, primary cells derived from the adult organism can be immortalized in vitro by the ectopic expression of hTERT. Age-dependent telomerase loss of function is believed to serve a tumor suppression mechanism, and telomerase is reactivated in many cancers [7,8]. Though cell cycle arrest and replicative senescence happen when telomeres become critically shortened [2,3,4], excessive shortening of the telomeres was shown to be tumorigenic, and short telomeres may increase genetic instability and tumor formation in mice [9,10].

**Figure 1:**
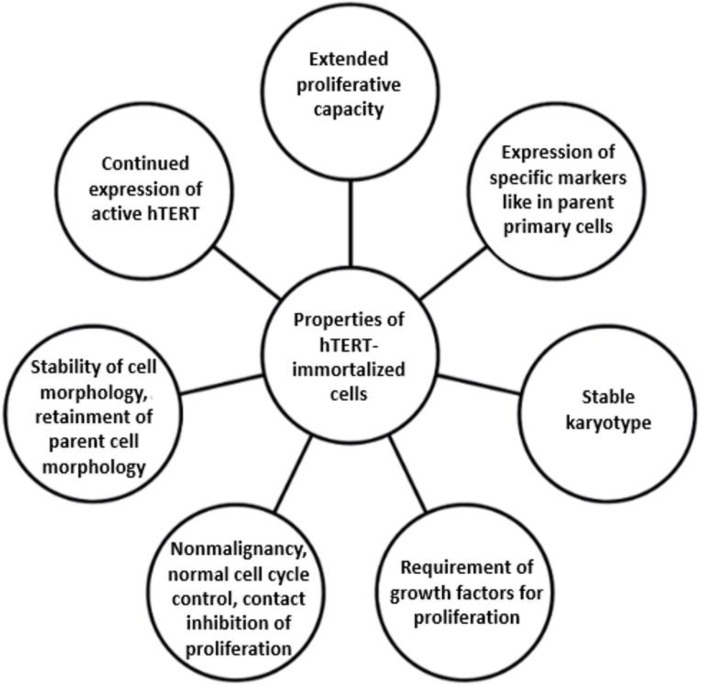
Characteristics of hTERT-immortalized cells.

The idea of immortalization consists of overcoming replicative senescence and obtaining cells possessing properties (except senescence) very close to their wild-type parent cells. Several criteria and tests are widely used for evaluating this. Immortalized cells have to retain a normal karyotype, not have malignant and tumorigenic properties, and divide not much faster and possess similar characteristics (marker expression, morphology, and phenotype) compared to the normal parent cells. Moreover, if cells are immortalized only via hTERT stimulation, they must differ from the ancestors in hTERT activity, and they have to gain their immortality through it and not via any obtained oncogenic mutations. The characteristics of cells after successful immortalization via the ectopic expression of hTERT are listed in Figure 1. A global biological resource center, the American Type Culture Collection (ATCC^®^, Manassas, VA, USA), produces immortalized cells and offers an hTERT-immortalized Cell Culture Guide [11], which contains information about different immortalization options and techniques. However, this information is general and contains no specific step-by-step description of the approaches for obtaining immortalized cells. Furthermore, a limited number of immortalized cell lines is represented in the ATCC catalogue, while researchers need a broader spectrum of immortalized cell lines for their work, the obtainment of which is complicated. In addition, there is no clear description of the criteria that cells should meet after immortalization. A certain list is given without specification of the parameters and boundaries of these criteria. All hTERT cell lines represented in the ATCC catalogue are tested for having extended proliferative capacity, a stable karyotype, selected phenotypic markers according to the tissue of interest, and the continued expression of hTERT. And if it is clear about the analysis of the karyotype stability, questions arise about the appropriate level of hTERT expression and the method which is the best to evaluate it. Similar problems occur when characterizing certain cells, since only one phenotypic marker is not sufficient, especially in the case when the traits of the cells may change significantly after immortalization. Furthermore, there are no ubiquitous specific markers for several types of cells, such as cells of mesenchymal origin (e.g., fibroblasts). Questions also arise about the rate of cell proliferation—to which extent its change is acceptable and when the tumor transformation occurs. There are no exact data about these characteristics. Different papers contain various opinions on their assessment and their acceptable range. At this point, the main concern of our paper is how to characterize immortalized cells to be sure that they became immortalized and, at the same time, did not become malignant.

## 2. Immortalization Approaches and Methodological Strategies

**Figure 2:**
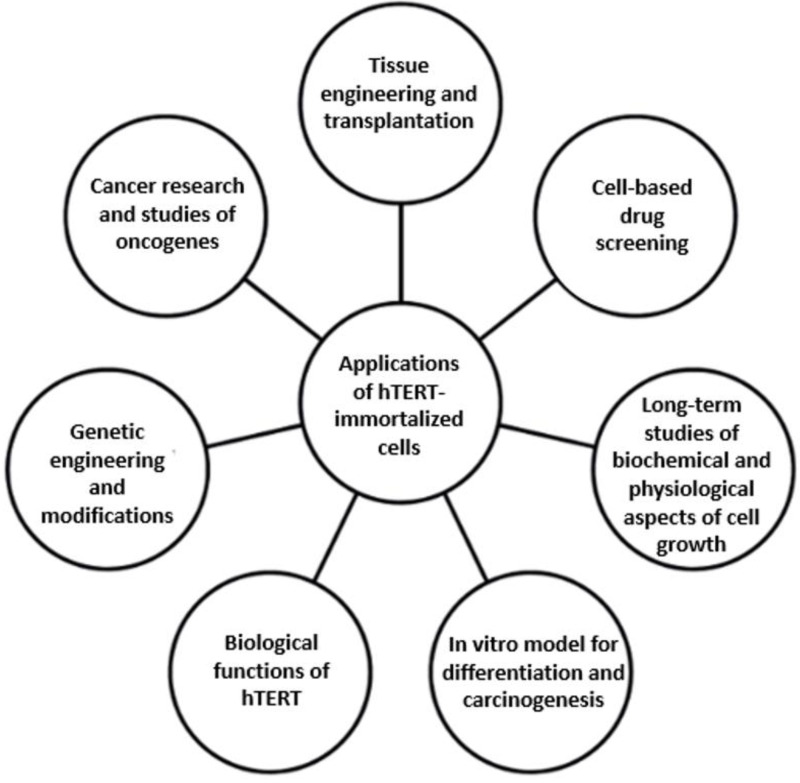
Applications of hTERT-immortalized cells.

Immortalized cells are a valuable tool in cell biology (Figure 2); therefore, the development of immortalizing techniques and methods for characterizing immortalized cells is very important. There are many situations in which the transient rejuvenation of cells is necessary, for example, when large quantities of cells are required for biochemical analysis, genetic manipulations, or genetic screens. Multiplying cells derived from limited human samples with hereditary diseases gives the opportunity to obtain enough material for investigation. The ectopic expression of hTERT has been shown to immortalize human skin keratinocytes [12], dermal fibroblasts [12,13,14], muscle satellite (stem) cells [15], vascular endothelial cells [16], myometrial cells [17], pigment retinal cells [18], and human mammary epithelial cells (HMECs) [19]. Moreover, human bronchial, corneal, and skin cells forced to express hTERT can be used to create organotypic (3D) cultures (bioengineered tissues) that express differentiation-specific proteins, demonstrating that hTERT by itself does not alter normal cell physiology. Implementing hTERT immortalization offers the possibility of producing tissues to treat a variety of chronic diseases and age-related medical conditions caused by telomere-based replicative senescence [20].

Cell types are different in the immortalizing methods that they demand and ways to characterize and test them after transformation [12,21,22,23,24,25,26,27,28]. Although only the ectopic expression of hTERT is usually sufficient for immortalization, sometimes it is not enough, and it is combined with one or more other immortalization agents, which depends on the particular type of cell. For example, genes encoding viral (simian virus 40 (SV40) large T antigen [21,22] and human papilloma virus 16 (HPV-16) E6/E7 [23] or non-viral (Cdk-4 and Bmi-1) oncoproteins [12,24,25] can be used together with hTERT. Nevertheless, the introduction of virus genes and oncoproteins has an important disadvantage: the cells usually obtain aberrant karyotypes, while hTERT-only immortalized cells usually maintain a stable karyotype. Thereby, using viruses and oncoproteins may lead to incomplete immortalization or oncogenic transformation [12,26]. There are different approaches that can lead to obtaining cells with different properties and characteristics. The karyotype and immortalization stability may depend on the method of choice [19,29,30]. The immortalization stability and cell properties have to be checked via long-term cultivation [13,14,31,32,33,34]. Detailed techniques, mechanisms, advantages, and disadvantages of different immortalization approaches were discussed in the review of Sutyagina et al. [26]. Table 1 summarizes resources for hTERT immortalization that were used in the studies that we review in the current paper.

The evidence of successful immortalization may not be immediate, because the cells experience stress after transformation and selection procedures, which may lead to slowing down the proliferation [13]. The purity of the cell culture is also important to consider. Therefore, the required cells have to be selected from the tissue cell mix before the transfection procedure. Otherwise, after obtaining immortalized cells which do not possess the required properties, it is difficult to distinguish whether they lost them because of the manipulations or because the wrong cell population has been transfected. Often, transfection occurs only in about 5–10% of cells; consequently, the next step is to select the necessary immortalized cells. Selection based on antibiotics is widely used and efficient but is toxic for the cells and may lead to the death of the immortalized cell population when it is small. In addition, antibiotic resistance genes potentially limit future application of the cells, if the researcher needs to introduce some other changes into them.

Selection using fluorescent markers is also possible if the immortalized cells contain a fluorescent label together with hTERT. This approach may replace selection with antibiotics, and the immortalized cells exhibit fluorescence, which is useful for some research. Nevertheless, required further fluorescence-activated cell sorting (FACS) can also lead to cell loss and cause mortality. However, visual assessment in flasks is not accurate and does not allow for the selection of the required cell population. Additionally, cell fluorescence can limit the possibilities of further staining for some scientific purposes.

When it is necessary to obtain immortalized cells only with hTERT and without any other insertion (i.e., antibiotic resistance or fluorescence genes), difficulties arise with the selection of the required cells. The most efficient approach in this situation is to culture them for an excessive time to ensure the elimination of non-immortalized cells, and it is impossible to do quickly. Therefore, the success of immortalization will not soon be evident. Complications arise when generating a stable cell line representing a homogeneous population derived from a single cell—the approach is called cloning, which is usually used to obtain tumor cell lines [33]. For this purpose, it is necessary to select single-cell clones, but this can result in selecting abnormal cells, since non-tumor cells are unable to grow alone without contact with each other [31]. Thus, the immortalized cells should simultaneously exhibit the properties of normal cells and have contact inhibition [29,32].

Consequently, it is important to take into consideration all these points when planning work and calculating the time required for obtaining the immortalized cells. Various aspects of these processes are discussed in more detail below.

## 3. Approaches for Evaluation of Immortalization

### 3.1. Proliferation Rate and Dividing Capacity

The absence of proliferation limitations and a stable growth rate without slowing down caused by cell senescence are the basic purposes of immortalization. Thus, in most studies, these capacities of the immortalized cells were tested (Table 2). A reliable approach is to culture immortalized cells for abundant number (50–70 or even 100 and more) of passages to be sure that they have not lost their multiplying capacity and original properties [8,13,14,31,32,33,34]. This is one of the best criteria, but it is lengthy, which is a possible reason why some investigators, to ensure immortalization, test the cells after culturing them only for 25–30 passages or even right after sorting [12,35,36,37]. Thus, this approach has disadvantages because certain primary cells, such as fibroblasts from young donors, might not decrease their proliferation rate for 30–35 passages, retain their original appearance, and begin to senesce only after the 40th passage. In some cases, after publishing the results of their work, the authors continue culturing the described immortalized cells to make them reach enough doublings for proving their proliferative capacity [13,31,38]. It is important to use wild-type parent cells as a control and culture them in parallel with immortalized ones to continuously compare them. Cells originating from another donor (if used as a control) might be different in growth rate, senescence dynamics, and other parameters [8,13,29,35].

It is also important to evaluate the proliferation rate. The cells may be grown for a long time, and the authors show their growth rate to stay unchanged compared to non-transfected cells [13,33] or do not show these data [31]. In some articles, the proliferation rate is evaluated but there is no information about the proliferation capacity [12,35]. Moreover, there are studies in which the proliferation rate and capacity are not assessed after a long culturing time [36,37].

The acceleration of the proliferation rate compared with that of the parent cells could be evidence of neoplastic transformation rather than cell immortalization. This case is described in the study of Xie and colleagues [29]. They performed the immortalization of fibroblasts via hTERT alone and hTERT with the SV40 large T antigen and observed two-times faster proliferation and chromosomal abnormalities in the latter, while the first one had a normal karyotype [29]. In another study, immortalized keratinocytes showed 13–20% acceleration of the proliferative rate (by 5-Ethynyl-2-Deoxyuridine labeling and flow cytometry) and a minor change in size and morphology [12]. However, the absence of neoplastic growth was demonstrated by the tumorigenic test via xenotransplants in immunocompromised mice. Furthermore, in this work, the proliferation rate was the same for immortalized fibroblasts on P30 compared with that of their wild-type reference on P15 (shown by immunocytochemistry staining (ICC) for Ki67). Thus, the rate of division is a matter of concern—which is still acceptable and which already exceeds the permissible values?—but may serve a warning about malignant transformation in some cases.

### 3.2. Karyotype Analysis

As a rule, cells immortalized with hTERT retain a diploid karyotype [8,31,32,38]. However, spontaneously immortalized cells (e.g., HaCaT, NIH-3T3) might become pseudo-diploid, especially at a high passage number, but they usually maintain the functions of the primary cells. In one of the first studies on immortalization with hTERT, after obtaining immortalized human fibroblasts and retinal pigment epithelial (RPE) cells, their chromosomes were shown to be normal by detailed G-banding [38]. Robertson et al. obtained the same result at the 63rd passage for corneal epithelial cells immortalized with hTERT [32]. On the contrary, using oncogenes for immortalization may lead to cell aneuploidy. It has previously been demonstrated that human fibroblasts immortalized via hTERT alone had a normal karyotype, while fibroblasts immortalized via hTERT combined with SV40LT acquired an abnormal karyotype including complex chromosome losses and multiple (>20) overlapped clonal abnormalities at the 55th passage [29]. Chromosomal defects were not surprising for the authors because the large T antigen is known to cause genomic instability and malignant transformation [30].

Nonetheless, in some papers, investigators do not mention karyotype analysis [12,13,33,34,36,37,39]. In an article about work on primary hepatic stellate cells immortalized with SV40LT, which is known to cause chromosomal abnormalities, the authors claim that these cells may be used to develop anti-fibrotic therapies [39]. Though the authors did not show information on the karyotype, they demonstrated the absence of tumorigenicity and analyzed the gene expression and functional characteristics of the immortalized cell line.

### 3.3. Cell Morphology and Phenotype

Generally, the shape, size, and appearance of immortalized cells have to stay unchanged and closely resemble that of the parent cells. It is clear that a completely different shape is not acceptable, for example, if after immortalization, elongated cells become rounded. However, there are no clear requirements for the stability of the cell morphology and the degree of its changes. As seen from the scientific literature, researchers usually estimate this approximately, by eye under a microscope [29,31,36,37,38]. In the study of Xie et al., fibroblasts transduced with SV40LT + hTERT became round and showed aggregation, the absence of contact inhibition, and anchorage independency in the soft agar assay—together with an abnormal karyotype [29]. Accordingly, the investigators decided not to use these cells in further studies and worked only with hTERT-immortalized cells. Pan et al. presented phase contrast and electron microscope pictures showing unchanged morphology of the immortalized with SV40LT hepatic stellate cells on the 30th and 100th passages [39]. They concluded that morphology of the immortalized cells did not differ from the parent wild-type cells but did not provide pictures of the latter in the article. Moreover, some authors do not pay attention to the appearance of the cells and provide no information about it in the papers [13,32,34,35].

Therefore, in the absence of common rules in the evaluation of the change in the morphology of the cells, researchers need to take advantage of current knowledge in cell biology to compare the cells before and after immortalization. In one of the articles, the authors performed a detailed morphological analysis of phase contrast microscopy before and after immortalization, and it included various parameters: area, perimeter, solidity, circularity, fit ellipse minor axis, and minimum caliper diameter [12]. The investigators also measured flow cytometry forward-scatter and side-scatter data to estimate and compare the size and granulation of the cells. They did not find a clear relationship between morphology and immortalization for each fibroblast parameter, although some cells had minor differences. The immortalized keratinocytes had smaller size and decreased granulation, which could be connected with their higher proliferation rate. Subsequently, the authors performed tumorigenicity testing and checked the retention of different characteristics of the immortalized cells compared to the parent cells, which confirmed that the changes were not critical.

### 3.4. Cell Senescence Evaluation by SA-β-Gal Staining

Normal cells senesce as the number of divisions increases and begin to acquire a set of phenotypic characteristics (visual senescence markers): an increase in the cell size and cytoplasm nuclear rate, cell flattening, irregular shape, etc. [40]. Cell senescence is accompanied by lysosome dysfunction, and in aging cells, lysosomal beta-galactosidase (SA-β-gal) increases its activity, which is used as a marker for replicative senescence in mammalian cells [41,42]. The chromogenic substrate 5-Bromo-4-chloro-3-indolyl-β-D-galactopyranoside (X-gal) stains cellular SA-β-gal and helps to determine senescent cells in cultures. Several articles report an increase in the SA-β-gal staining level of the control parent cells near senescence compared with hTERT- immortalized cells [12,13,35,38] (more details are presented in Table 2). However, most researchers do not analyze immortalized cells for senescence markers [13,14,29,31,32,33,34,36,37], probably because they consider it an optional test. We suppose that the more characteristics the authors evaluate, the more accurate and reliable the results will be. It is important to compare the SA-β-gal staining of immortalized cells with the control parent cells at early passages without aging traits and at later passages when the parent cells become senescent.

In another study, the authors did not observe any difference between immortalized keratinocytes and their ancestor primary cells in SA-β-gal staining [12]. They explain the results by the drawbacks and limitations of this method, such as false positive cases due to the serum starvation of cells, the composition of the medium, or more complex processes involving lysosomal function. The activity of SA-β-gal might also depend on the DNA synthesis and proliferation rate, which are other factors correlated with the senescence of cell cultures [43]. Additionally, a recent paper reported an observer bias and the lack of standardization with respect to it [13]. One of the possible ways to overcome this drawback is applying a highly sensitive quantitative fluorescent β-Galactosidase assay (MUG) to measure complete endogenous SA-β-gal activity in cell lysates [42]. Thus, as the SA-β-gal approach may be accompanied by false positive/negative outcomes, more precise methods for senescence evaluation need to be developed. Perhaps it would be promising to use the fluorophore-conjugated Sudan Black-B analog (GLF16), suitable for in vitro and in vivo analysis, which has recently been proposed by Magkouta et al. [44].

### 3.5. Expression of Specific Markers

It is essential to prove that immortalized cells retain the expression of the same specific markers as their primary parent cells [12,31,32,33,34,35,37] (more details are presented in Table 3). In addition, it is possible to sort cells after immortalization according to their marker expression using a serial dilution method to choose only those which have the markers of the original culture [31]. The problem exists for cell types like fibroblasts, which have controversial specific markers. Evtushenko et al. performed immunofluorescence (IF) staining for collagens type I, IV, and VII; fibronectin; and fibroblast-specific protein 1 (S100A4) to show that immortalized fibroblasts expressed the same markers as their wild-type ancestors [12]. Nevertheless, some authors considered the evaluation of only vimentin in fibroblasts to be sufficient [37]. In hTERT-immortalized foreskin fibroblasts, the expression of PDGFRβ (platelet-derived growth factor receptor beta) can be evaluated, as PDGFRβ is required for the development of mesenchymal cell types and plays different roles in the function of fibroblasts, tissue homeostasis, and regeneration [45]. For hTERT-immortalized epithelial cells, the recommendation of ATCC is IF staining for pan-cytokeratin [11]. There is also an approach to assess the expression of a specific set of genes at the mRNA level, which seems quite accurate and quantitative. For scleroderma fibroblasts, Kapanadze et al. analyzed the mRNA levels of nine profibrotic genes including COL1A1, TSP1, CCN2, Smad1, PLOD2, fibrillin 2 (FBN), collagen XI, desmin, and tenascin C by qRT-PCR [33]. However, not all studies evaluate immortalized cells for specific markers [13,14,29,36].

### 3.6. Telomer Length (TL) Assessment

TL is an important parameter of the proliferative potential of cells. Cell immortalization via the activation of hTERT has to lead to an increase in TL; thus, TL is a prominent marker of immortalization. Nevertheless, this parameter is rarely assessed [13,14,35,38,46] (Table 2), which could be related to the complexity of the methods for its evaluation and differences in measured characteristics. The average TL has been measured by a monochrome multiplex quantitative PCR method (MMqPCR) [47]. Another approach is the Telomere Shortest Length Assay (TeSLA) method [48], helping to measure the average TL and providing quantitative information on the shortest telomeres (<1.6 kb). The use of the TeSLA (compared to average TL measuring) is based on the observation that the emerging shortest telomeres activate DNA damage responses to switch on cell cycle arrest, leading to cell senescence [49].

The elongation of TL was shown in hTERT-expressing cells after immortalization, while telomeres of control cells shortened in the course of senescence [13,38]. Transient immortalization with hTERT resulted in the elongation of the shortest telomeres of the fibroblasts from donors with Hutchinson–Gilford progeria syndrome [46], which is characterized by accelerated telomere erosion [50]. The authors did not observe a significant increase in the average TL in hTERT mRNA-expressing cells, but they detected the extension of short telomeres. This is important to consider in studies where TL measurements do not show average telomere elongation in immortalized cells. Another study on hTERT immortalized Hutchinson–Gilford progeria fibroblast cells demonstrated that the main elongation effect of ectopic hTERT expression occurs during the early period (20 population doublings) after immortalization, with only moderate further telomere elongation or even TL reduction [13]. This initial telomere stabilization and elongation leads to a long-lasting replication induction, the prevention of senescence, and phenotype improvement. However, this confirms the idea that the stabilization of the telomere cap (and less the total length of the telomeres) is responsible for the positive effects on cell proliferation and rescuing the juvenile phenotype.

The important point is that TL depends on cell type. Demanelis et al., using postmortem human samples of more than 25 tissue types, performed an extensive study on variability in TL across tissue types and the influence of aging, ancestry, genetic variation, and other biologic processes on TL [51]. Among other conclusions of this work is that relative TL (telomere repeat abundance in a DNA sample relative to a standard sample) is inversely associated with age in most tissues, and this association is strongest for tissues with a shorter average relative TL. As long as many parameters influence the variability of TLs, which can make it difficult to compare them and perform appropriate interpretation of the results obtained in different studies and cell lines, TL does not seem to be a reliable parameter for assessing cell immortalization but rather an additional one.

### 3.7. hTERT Expression Level (mRNA and Protein)

The level of hTERT expression is an important characteristic of cells immortalized via telomerase reactivation and is expected to increase after immortalization. Regulation of the expression is a complex multifactorial process, so it is necessary to assess hTERT at both the mRNA and protein levels. An epigenetic mechanism regulating hTERT expression has been previously demonstrated [52], and different and multilevel pathways were shown to be involved in the telomere control in the cell [46]. One or a few molecules of the hTERT catalytic unit can force telomere elongation; therefore, changes in the expression levels of hTERT measured by standard methods may not indicate telomerase activity. Additionally, hTERT may be present but does not work or may be involved in other functions, which adds complexity to interpretation of the results. Several naturally occurring alternatively spliced variants of the telomerase were reported, which had no telomerase activity but stimulated cell proliferation [53]. The increase in hTERT protein expression detected by ICC without overexpression of its gene indicates that post-transcriptional regulation of hTERT occurs [37]. In cancers with high telomerase activity, hTERT expression was detected in almost all neoplastic cells, and its intensity was correlated with telomerase activity levels, and cells with telomerase activity had nuclear positive signals [36].

When assessing hTERT expression at the mRNA level, it is important to consider that using primers for various hTERT sequences can produce different results. Often, primer pairs are designed to the reverse transcriptase domain (RT domain) of TERT (exons 5–10), while exons outside of the RT domain are not measured (i.e., exons 1–4 and 11–16) [52]. This explains why hTERT transcripts can be detected in various telomerase-negative cells and tissues, but the resulting mRNA is not full-length mRNA, and it is unable to produce active telomerase [53]. The expression of catalytic and RNA subunits of hTERT was shown to be low or undetectable in most tissues of the human body and not associated with TL within any tissue, as was shown in a large-scale study [51]. The exception was the testes, as TERT was expressed in all samples at a higher level, and their telomeres were longer, obviously, because testes contain spermatogenic cells with high telomerase activity. It was shown that the hTERT mRNA expression level was increased and remained elevated after the immortalization of fibroblasts from healthy donors and donors with Hutchinson–Gilford progeria (HGP) syndrome. In addition, there was no positive correlation between hTERT mRNA and TL in the primary control, but the authors observed a weak inverse correlation in HGP cells, consistent with the hypothesis of mRNA downregulation in cells with long telomeres [13].

One of the main questions concerns hTERT localization and its staining via ICC and IHC (immunohistochemistry). Problems with antibody quality, specificity, and performance arise during the analysis of hTERT at the protein level. Moreover, the specifications of antibodies from various manufacturers contain different images of hTERT localization in the cell. The general consensus is that it should be located in the nucleus [12,36,54] and, during cellular stress, in the mitochondria. Nevertheless, in some papers, cells had dot-like staining in the cytoplasm [36,37], which is contradictable as cytoplasmic hTERT may have functions distinct from its telomeric repeat-adding function in the nucleus. In studies on the clinical application of anti-hTERT antibodies, ICC staining of urine samples from patients with urothelial carcinoma was considered positive only when the nuclei stained [54,55]. Moreover, cytoplasmic staining was not regarded as a positive result. Lymphocytes were used as an internal positive control, and nonurothelial components, such as bacteria, lymphocytes, and neutrophils, consistently stained positive.

Western blotting (WB) is also used to measure hTERT expression level [12,29,36]. More intense staining of the hTERT band was shown in all immortalized fibroblasts and keratinocytes compared to the HaCaT cell line, while almost no staining in primary cells was detected [12]. Nonetheless, the authors did not perform quantitative analysis of the WB, which gives more accurate results because visual assessment is very subjective [12]. WB analysis confirmed IHC results, showing that hTERT is expressed not only in the nuclei but also in the cytoplasm of cancer cells [56]. It is important to use positive and negative controls for hTERT protein levels in the WB method. The fibrosarcoma HT-1080 human cell line has a high level of hTERT expression, while SW13-ALT cells are telomerase-negative immortal fibroblasts; therefore, they may be used as controls [36]. It was shown that normal fibroblasts transfected with hTERT had a strong band in WB, but it had a lower intensity than the band of HT-1080 cells. These data were also assessed visually without a quantitative analysis of the WB. When analyzing the figures in the paper, it seems that the intensity of the bands is the same in both cell lines, which indicates that the protein level in the immortalized cells is similar to that of the tumor cell line. This is a controversial result because the immortalized cells are expected to have lower protein activity than tumor cells. In our opinion, to make the results complete, the level of hTERT protein in primary fibroblasts before transfection with hTERT has to be evaluated. We consider this control very important and necessary.

In summary, data on hTERT expression levels obtained in studies are very different (Table 4). hTERT expression is frequently assessed, but researchers rarely (or even never) perform all types of analysis: the evaluation of RNA expression and total protein by WB and ICC staining to localize the protein in the cell. Often, the authors do not present information on the passage of the cells (or the number of population doublings) at which they performed analyses, which is important for understanding the results and the unification of methods, as we discuss in different sections of the current paper. Sometimes, the articles do not provide information about the manufacturer of the antibodies and the dilutions of antibodies used for the studies, although it is important, considering the small amounts of hTERT in cells. We suppose that this may lead to an underestimation of the hTERT amount in wild-type cells, which could be lower than the detection level of the method. More accurate and quantitative characterization approaches may be helpful in this concern. That is why, though the level of hTERT expression is an important characteristic of immortalized cells, we propose that assessing hTERT expression level may be optional and it is better to evaluate hTERT activity.

### 3.8. TRAP

The telomere repeat amplification protocol (TRAP) is a PCR-based assay developed three decades ago and still used for the routine evaluation of telomerase activity [57]. The principle of the method is that a substrate (TS primer) for elongation by telomerase is added to the lysate of the studied cells. Telomerase extends this primer, and then a polymerase chain reaction (PCR) produces a hexamer ladder of extended products (so-called TRAP ladder), which are visualized on a polyacrylamide gel [29,35,36]. With the generation of droplet digital PCR (ddPCR) technology [58], the TRAP gained a modification, which made it faster, more precise, and reproducible and excluded the step of using polyacrylamide gel electrophoresis (PAGE). In recent years, many studies [12,14,32,33,34] have used the TRAPEZE^®^ RT Telomerase Detection Kit [59]. As technology developed, several modifications appeared, and it became quantitative with the use of fluorescence energy transfer primers to generate fluorescently labeled TRAP products, which permit the accurate and sensitive analysis of telomerase activity.

Telomerase activity is supposed to be absent in most primary cell cultures derived from adult specimens. In tumor cells and immortalized cells with artificially added hTERT, its activity has to be detected by the TRAP [57]. It is important to use reference samples when implementing this approach [12,36]. As a negative reference, it is better to use wild-type parent cells before immortalization, and for the positive reference, any cancer cell line with known elevated hTERT activity. In an ideal situation, hTERT activity has to be higher than in wild-type control cells and lower than in a cancer reference. When analyzing the results of articles from Table 2, it can be concluded that there are no established criteria for what the difference in activity should be, and the researchers judge subjectively and based on experience. For example, it is unclear whether it is normal when the hTERT activity of the immortalized cells is higher than that of the control tumor cells or whether this can be a sign that the immortalized cells are undergoing neoplastic transformation. Bodnar et al. used the TRAP with the gel visualization modification to measure telomerase activity in immortalized RPE and fibroblast clones against the human tumor cell line H1299 as a positive control [38]. They showed that the activity ranged from 65 to 360% in the RPE clones and from 86 to 95% in the fibroblast clones. It is reasonable to perform the TRAP at early, middle, and late passages after immortalization (40 to 264 population doublings, passages 10–63), as it was performed in the study on the immortalization of primary human corneal epithelial (HCE) cells [32]. The researchers showed constant telomerase activity at different time points using human mammary epithelial (HME50-5E) cells as a positive control. Normal HCE cells were TRAP-negative. However, despite the importance of this parameter for hTERT-immortalized cells, not all studies evaluate telomerase activity by the TRAP [13,31,37].

Another point that may be emphasized is that considering the nuclear telomeric activity of hTERT, it is better to perform the TRAP using nuclear and not cytoplasmic or whole-cell extracts. In the study of Kyo et al., TRAP assays confirmed significant telomerase activity in both the nuclei and cytoplasm of cancer cells [56]. The authors performed a careful evaluation of cytoplasmic and nuclear fractions of different cancer cell lines using WB and the TRAP; they found hTERT activity and staining in the cytoplasm as well and concluded that hTERT in the cytoplasm may have some unknown function. Additionally, they showed that hTERT expression does not strictly reflect telomerase activity in cancer cell lines.

There are several concerns regarding the TRAP application. Different variants of the method have been used in studies: the TRAPEZE^®^ RT Telomerase Detection Kit, self-collecting all the reagents to perform analysis [60], the semi-quantitative evaluation of the fragments on gel (TRAP ladder) [29,35,36,38], etc. There is the implementation of various measurement units, which makes it difficult to compare data reported in papers. The lack of negative and positive controls leads to complications in assessing the reliability of the presented results from the studies. In addition, during the TRAP assay, PCR priming errors and PCR bias may occur for short fragments [58].

### 3.9. Tumorigenicity Evaluation

The main tests for assessing the tumorigenicity of cells are the soft agar colony assay [61,62], in vivo methods [12,29,35], contact inhibition [32,63], and serum-free growth [11]. Nonmalignant cells must also retain their properties of normal cell cycle control (functional p53 and pRB checkpoints) [24], the requirement of growth factors for proliferation [64], and normal growth responses to serum and mitogens [11]. Most of the concerns about tumorigenicity testing for clinical use have been actively discussed in recent years (more with respect to human pluripotent stem cells) [65,66]. Many methods produce relative results and may not be effective for the proper assessment of the tumorigenicity potential of immortalized cells. In the context of clinical implementation, the evidence of cell senescence after serial passages in vitro is considered proof of the cells being nontumorigenic. This cannot be applied to immortalized cells because they must have unlimited proliferation potential.

The in vivo test using immunodeficient mice (e.g., SCID/NOD) is the most reliable but expensive and labor-intensive [66]. In addition, there are several concerns for testing human cells in mice. The tested cells are injected into immunodeficient animals where they may grow more readily, and their features may differ in this microenvironment compared with the human organism, possessing various immune responses [63]. The alternative and faster in vivo method is testing in a chicken embryo, for which the incubation period is only 3 days, although the same problems arise utilizing this approach as in the case with mice.

Other methods to evaluate tumorigenicity are less evidence-based and suitable for clinical implementation. Nevertheless, if immortalized cells are not supposed to be used in clinical practice, it is better to perform any. The protocol based on using soft agar for cell growth (soft agar colony formation assay) is time and labor-consuming (including 20–30 days of incubation) and skill-demanding [61]. An improved version of this method is more accurate and sensitive, implementing an image-based screening system built upon a high-content cell analyzer, which allows detecting only one tumor cell in approximately 10,000,000 diploid cells within 30 days [62].

In all of these methods, HeLa cells are used as a positive control [12,61]. This cell line has been widely used in laboratory studies, but it is cancerous, and its proliferation rate is very high. Thus, comparing cells of interest with HeLa can lead to false negative results because many other cell lines, including cancer and especially primary cell lines, which are also often used as a negative control, divide more slowly. Accordingly, it is important to choose the correct positive control that will correspond closely to the properties of the primary cells that are immortalized. For example, one applicable option is to choose tumor cells of the same tissue and origin.

Immortalized keratinocytes with an elevated proliferation rate were transplanted into the testes of immune-deficient mice (as testes are among the immune-privileged organs) [12]. Interestingly, several cells from the sample survived after 10 weeks in the testes but did not form any tumor. Mice in the control group that received HeLa cells developed tumors. Human hepatic stellate cells immortalized only with SV40LT did not form colonies in mice either, though they had an elevated growth rate and could be cultured in medium with only 1% of fetal bovine serum (nevertheless, they did not grow completely without serum) [39]. These observations suggest that the increased cell proliferation of immortalized cells does not necessarily mean their tumorous transformation. Nevertheless, tumorigenicity is an important parameter, and it has to be assessed together with other characteristics that were described above. However, not all researchers investigate this feature in their studies [14,31,33,34,36]. More information from different papers is collected in Table 2.

## 4. Discussion

As discussed above, there are various immortalization approaches, which can result in obtaining cells with different properties. Using only hTERT was shown to produce cells with characteristics closer to wild-type parent cells. Thus, it is important to note that the immortalization approach influences karyotype stability, the properties that the cells obtain, and the efficiency of immortalization itself.

**Figure 3:**
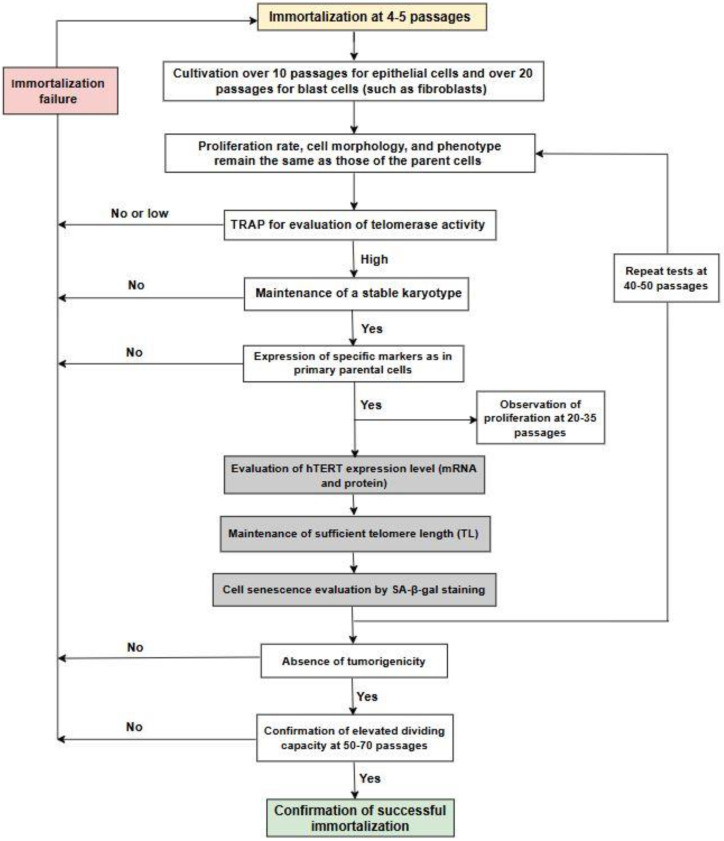
Verification steps for evaluation of hTERT-immortalized cells. Grey boxes indicate steps that may be omitted.

Cells may become stressed immediately after immortalization, and it is necessary to wait and observe the culture to assess the success of immortalization. If immortalization is performed soon after the isolation of the cells from the tissue, there is a possibility of obtaining a heterogeneous culture, and immortalization may result in a mixed population of cells with unknown composition. Therefore, it is important to select cells before immortalization by culturing them in a selective medium or by specific sorting. If there is an intension to use cells after the immortalization procedure in clinical practice, there is a necessity to de-immortalize them for safe introduction into the body, and immortalization reversibility in the context of cell therapy biosafety needs to be considered [26]. In the current paper, we discussed multiple approaches for the evaluation of immortalization success and the characteristics of immortalized cells. Our opinion on the steps and their sequence for the verification of immortalized cells is summarized in Figure 3. First, at 5–10 passages after immortalization, it is necessary to check the proliferation rate, cell morphology, and phenotype, which have to stay the same as those of the parent cells before immortalization. Next, it is important to evaluate telomerase activity because it is used as an immortalization agent. The subsequent steps include verification of the karyotype stability and confirmation of the expression of the same specific markers as in the primary parent cells to verify that no tumor transformation has occurred. Only a long period of cultivation shows an extended dividing capacity of the immortalized cells. Additionally, it is important to check cell characteristics again after 40–50 passages to confirm immortalization stability and the required cell properties. It is reasonable to evaluate tumorigenicity after all these procedures, especially if testing in mice is used, since it is time-consuming and expensive. Grey boxes in the scheme indicate steps that may be omitted, as their results may be controversial and do not provide clear confirmation of immortalization.

Immortalization has been in the scientific field for at least 30 years. Therefore, we could not evaluate all the corresponding articles, but we examined a broad range of different data from a long period. A brief overview of these papers is given in Table 2, where the main characteristics of the immortalized cells are presented and compared. It is important to note that none of the studies presented an evaluation of all the characteristics of immortalized cells at once. Usually, only 3–4 parameters were assessed, and this does not demonstrate a full picture of immortalization and its final success. Moreover, not all papers have paid sufficient attention to such important and relatively easy-to-measure parameters as the karyotype, stable and long proliferation, and cell shape retention. Resulting tumorigenicity testing, especially in vivo, is also very important. Nevertheless, only some investigators perform it because of its complexity and expensiveness, although it is impossible to confirm the absence of tumorigenic properties without this testing.

## 5. Conclusions

Immortalization approaches play an important role for cell science, for investigations of inherited diseases, and for biotechnology and biomedicine. These techniques have been used for a long time, but still there is no established list of methods and parameters for the evaluation of immortalization success and the properties of the obtained immortalized cells. The future development of immortalization approaches is impossible without the elaboration of a precisely defined methodology, specifically, if immortalization is aimed at application in cell therapy and biomedicine. We hope that the questions raised in our paper will draw the attention of the scientific community and lead to a discussion and a search for solutions.

## Figures and Tables

**Table 1:** Resources for hTERT immortalization.

Reference	Cell Type	Reagents and Methods
[12]	normal and RDEB Fibroblasts and keratinocytes	Lentiviral transduction was performed by one or both vectors encoding hTERT cDNA and bmi-1 cDNA. Lentiviral transduction was followed by cell selection by their growth with the presence of puromycin. The hTERT cDNA sequence was amplified from Addgene plasmid #69809. A lentiviral plasmid (pGKloxPAzuriteTurboFP635 NLS) was constructed in the similar way except for using the Azurite insert instead of hTERT, which was amplified from Addgene plasmid #36086. The plasmid for BMI-1 expression was Addgene plasmid #12240.
[13]	Hutchinson- Gilford progeria syn drome (HGPS) fibro blasts, healthy control fibro lasts	All fibroblasts underwent infection procedures with a virus supernatant containing the hTERT-pBabe Puro vector from the packaging cell line PA317 in the presence of 4 μg/mL polybrene (Santa Cruz Biotechnology, Dallas, TX, USA, Cat. SC-134220) for two days. The vectors and packaging cell line were a gift from Dr. Woodring Wright (UTSW Medical School, Dallas, TX, USA). Following virus infection, 1 μg/mL puromycin (GIBCO, Thermo Fisher Scientific, Waltham, MA, USA, Cat. A11138) was used for four days to remove uninfected cells and acquire virus-infected cells.
[31]	hepatocyte-like hybrid cells	The pRRLsin.hCMV hTERT lentiviral expression plasmid (the kind gift of Dr. Noriyuki Kasahara, Department of Medicine, UCLA, Los Angeles, CA) was used. The telomerase vector was produced by three plasmid transient transductions of the self-inactivating (sin) hTERT lentiviral expression plasmid, 10 μg of the gag/pol plasmid, pCMV delta 8.2, and 2 μg of the envelope plasmid, pCMV VSVG, in a calcium phosphate transduction protocol according to the manufacturer’s directions (Clontech).
[29]	foreskin fibroblasts	pBabe-hygro-hTERT and pSG5-LT plasmids were from Addgene. All restriction enzymes and Phusion high-fidelity DNA polymerase were from New England Biolabs. The plasmids 5′-PTK-3′ and hyperactive piggyBac transposase (PBase) were kind gifts from Allan Bradley (Wellcome Trust Sanger Institute, Hinxton, United Kingdom). A 2.5-kb fragment of hTERT was released from pBabe-hygro-hTERT with EcoRI and BamHI and ligated into pSKCAG (termed pSKCAGhT1). The coding fragments of hTERT (hT2), enhanced green fluorescent protein (EGFP), and SV40LT were amplified from pBabe-hygro-hTERT, pEGFP, and pEGFP-N3 (Invitrogen).
[35]	AE pre-leukemia cell (fusion protein AML1- ETO)	Retroviral vector-expressing hTERT (pBabe-puro-hTERT) and a control empty vector (pBabe-puro) were obtained from Dr. Robert Weinberg (Addgene). Independent AE clones stably expressing hTERT or pBabe were selected through puromycin resistance. To produce the retrovirus, 1.5 µg of each construct was transfected, respectively, into phoenix-gp cells on 6-well plates along with pSV-ampho-env (1.0 µg) and pEQ-PAM3(-E) (0.5 µg) using TransIT reagent (Mirus, Marietta, GA, USA).
[33]	normal and scleroderma (SSc) fibroblasts	An EcoRI fragment containing the Kozak consensus sequence and the coding sequence of hTERT were obtained from the recombinant plasmid pLPC-hTERT (Clontech, Mountain View, CA, USA). The Entry clone pENTR1A-hTERT was constructed by inserting the hTERT EcoRI fragment in EcoRI sites of pENTER1A vector (Invitrogen, Carlsbad, CA, USA). Using Gateway technology, we introduced the hTERT gene from pENTR1A-hTERT into the pLenti4/V5-DEST vector to create the expression plasmid (Invitrogen). Lentivirus was made by transfecting pLenti4/V5-hTERT expression plasmid and viral packaging vectors (Packaging Mix, Invitrogen) into 293FT cells (Invitrogen) by lipid transfection (Transfectin, Bio-Rad, Hercules, CA, USA).
[34]	human mesenchymal stem cells (hMSCs)	A RevTRE-hTERT plasmid was constructed by ligation of the hTERT gene (kindly provided by Dr. Robert Weinberg Whitehead Institute for Biomedical Research) into the multiple cloning site of the RevTRE vector (Clontech). RevTRE-hTERT and RevTet-on (Clontech) plasmids were stably transfected to the PT67 packaging cell line using lipofectamine 2000 (Invitrogen, Carlsbad, CA, USA; 1.6 mg/mL DNA) to generate the recombinant viruses. Stably transfected packaging cells were selected with hygromycin or G418, respectively.
[32]	human corneal epithelial cells (HCE)	Myeloproliferative sarcoma virus MPSV-hTERT vector was cloned into a retroviral vector (pBABE-puro). Retroviral vector particles were generated by transfecting 30 μg of vector DNA into PhoenixE cells with a FuGENE® Transfection Reagent (Promega, Madison, WI, USA). Supernatants collected 48 h after transfection were used to infect the amphotrophic packaging cell line PA317 (CRL-9078; American Type Culture Collection, Manassas, VA, USA). After 7-day selection with puromycin, supernatants containing the retroviral particles were harvested for infections. Infecting medium containing polybrene was added, and the cells were cultured overnight.
[14]	skin fibroblasts from normal and ataxia telangiectasia (AT) individuals of Japanese origin	An EcoRI-BamHI hTERT cDNA fragment containing the Kozak consensus sequence and the coding sequence of hTERT franked by the restriction sites were obtained by PCR with pLXSN-hTERT (a kind gift from Dr. Denis A. Galloway (FHCRC, Seattle, DC, USA)) as a template. The retrovirus vector pCLXSN-hTERT was constructed by inserting the hTERT EcoRI-BamHI fragment between the EcoRI and BamHI sites of pCLXSN20 (Imgenex Corp., San Diego, CA, USA). The preparation of hTERT- and LXSN-retroviruses and infection protocols have been described previously. After the transfection of these retrovirus vectors, G418-resistant cells were selected.

**Table 2:** Immortalization characteristics.

Reference	Cell Type	Immortalization Agent	Proliferation	Karyotype	Morphology	SA-β-gal Staining
[13]	Hutchinson– Gilford progeria syn drome (HGPS) fibro blasts, healthy control fibroblasts	hTERT	increased growth rates following the period of stagnation for at least additional 300 days, or 50 PDs without apparent signs of senescence; a tendency for growth acceleration at higher PDs in some cell lines	n/d *	n/d	at high PDs, the average SA-β-gal values of all virus-infected HGP and control cells significantly reduced compared to primary cells at high PDs
[12]	normal and RDEB fibroblasts and keratinocytes	hTERT and hTERT plus BMI-1	no correlation between the percentage of Ki-67-positive cells in primary and immortalized fibroblasts; immortalized keratinocytes have increased proliferation compared to their primary states (shown by 5-Ethynyl-2-Deoxyuridine (EdU) labeling and flow cytometry)	n/d	analysis of phase contrast microscopy (area, perimeter, solidity, circularity, fit ellipse minor axis, minimum caliper diameter), flow cytometry forward scatter (FSC) and side scatter (SSC)	immortalization fibroblast staining intensity became closer to primary fibroblast from a young donor and, in most cases, significantly lower than in the paired primary cells; no changes between primary and immortalized keratinocytes
[37]	periodontal ligament fibroblasts (hPLF)	hTERT	at the 20th passage, hPLF-hTERTs maintained their proliferation rate and hPLFs senesced	n/d	at the 20th passage, hPLFs were in senescence, while hPLF-hTERTs maintained their morphology characteristics	n/d
[31]	hepatocyte-like hybrid cells	fusion of hTERT multi-lineage progenitor cells with primary hepatocytes	cells have been cultured for at least 12 months, no exact information about proliferation rate	no numerical or structural chromosomal abnormality	cobblestone culture pattern with round, square, and trapezoidal shaped cells; individual cells mainly mononuclear or multi-nuclear with 2, 3, or 4 nuclei	n/d
[29]	foreskin fibroblasts	hTERT	steady growth rate over 140 days (40 passages), parental fibroblasts grew slowly and became senescent at passage 10	normal diploid chromosomes	contact inhibition and anchorage dependency	n/d
[29]	foreskin fibroblasts	hTERT + SV40LT	over 140 days (100 passages), twice higher than the rate of parental and hTERT fibroblasts	abnormal karyotypes including complex chromosome losses and multiple (>20) overlapped clonal abnormalities	round cells, aggregation, and absence of contact inhibition and anchorage independency in the soft agar assay	n/d
[35]	AE pre-leukemia cell (fusion protein AML1- ETO)	hTERT	control cells grew at a rate of about 2 population doublings per week and stopped proliferating at around week 26; AE-hTERT cells showed continuous proliferative capacity at an enhanced rate of about 2.5 population doublings a week	leukemic AE-hTERT cells showed chromosome translocation t(3;7)(q27; q22)	the authors do not describe; the reader may guess by photos only	AE-pBabe cells had increased senescence compared to AE-hTERT cells at week 26
[33]	normal and scleroderma (SSc) fibroblasts	hTERT lentivirus	hTERT cells growth rates were unchanged compared to non-transfected cells; two clones passed M2 or crisis for >200 PDs; one of these clones entered crisis after 115 PDs but recovered to continue proliferation; another clone passed > 200 PDs without entering crisis and bypassed M2 entirely	n/d	more than 90 PDs without any apparent change in morphology	n/d
[34]	human mesenchymal stem cells (hMSCs)	inducible hTERT expression by the addition of doxycycline (i-hTERT hMSCs)	90 weeks; I-hTERT hMSCs expressing hTERT were able to proliferate at least 120 PDs, whereas i-hTERT hMSCs with turned off hTERT expression senesced at 30 PDs (as expected for wild-type cells)	n/d	n/ed **	n/d
[32]	human corneal epithelial (HCE) cells	hTERT	normal cell cycle kinetics at more than 240 PDs	normal karyotype and loss of p16 after passage 10	n/ed	n/d
[14]	skin fibroblasts from normal and ataxia telangiectasia (AT) individuals of Japanese origin	hTERT	control cells with vector alone reached complete senescence before PD 50; all the cells with hTERT continued to grow beyond PD 200 without reduced growth rate; both normal and AT immortal cells exhibited an apparent inhibition of growth as original primary cells when they reached confluence	diploid range with a modal number of 46	cells transfected with hTERT continued to grow beyond PD 200 with no indication of senescence like a flat shape	n/d
[36]	BJ foreskin fibroblasts	hTERT	n/d	n/d	normal	n/d
**Reference**	**TL**		**hTERT Expression**	**TRAP**		**Tumorigenicity**
[13]	mean TL nearly doubled in healthy cells; HGP cells showed markedly increased TL; telomeres elongated with a short delay of some PDs after immortalization, indicating that telomerase efficiently and rapidly elongated telomeres		high hTERT mRNA levels induced shortly after the infection, no correlation of growth rate with mRNA; expression reduced at higher PDs, cells continued growing at high proliferation rates	n/d		n/d
[12]	n/d		protein WB positive, ICC positive staining for some cells	increased telomerase activity by TRAPEZE kit–qPCR; in immorta lized cell lines comparable to the common immortal cell lines: 3T3 (NIH), HeLa, HaCaT; no telomerase activity in the primary cell lines		non-tumorigenic immortalized keratinocytes transplanted into Nude (NU/NU) mice testes
[37]	n/d		increased in hTERT protein expression (ICC) with no change of RNA	n/d		n/d
[31]	n/d		fluorescent ICC	n/d		n/d
[29]	n/d		confirmed by qRT-PCR and WB	strong telomerase activity by TRAP ladder on gel		no teratoma formation in immunodeficient mice
[29]	n/d		confirmed by qRT-PCR and WB	strong telomerase activity by TRAP ladder on gel, more than in hTERT fibroblasts		no teratoma formation in immunodeficient mice
[35]	Southern blot analysis; the immortalization of AE cells was not associated with telomere lengthening; AE-hTERT cells showed a progressive decline in TL similar to control cells; TL was eventually stabilized at about 3–4 kb, comparable to the TL of Kasumi-1 cells		n/d	telomerase activity was upregulated in AE-hTERT cells (becoming comparable to the levels in Kasumi-1 cells) by TRAP ladder on gel		AE-hTERT cells did not show features of stepwise transforma tion, with no leukemoge necity evident upon initial injection into immunode ficient mice
[33]	n/d		n/d	six base pair DNA repeats by TRAPeze kit and TRAP ladder on gel indicate that hTERT is overexpres- sed; non-infected normal dermal fibroblasts for a negative control		n/d
[34]	n/d		very high hTERT expression observed by RNA RT-PCR of RNA in i-hTERT MSCs induced with dox, while negligible expression was seen in non-induced and control cell lines	active telomerase enzyme in induced i-hTERT hMSCs by TRAPeze XL Telomerase activity assay; background level of telomerase activity in non-induced i-hTERT and wild type hMSCs, much higher activity level in induced i-hTERT hMSCs		n/d
[32]	n/d		n/d	TRAP-eze Kit-TRAP ladder on gel; telomerase activity was assessed at different passages, which correlated with 40 to 264 population doublings (passages 10–63); at all time points examined, hTCEpi cells had a positive TRAP assay for telomerase activity		n/d
[14]	TL determined by TRF length analysis was longer in the hTERT-transfected cells than in the original cells		n/d	TRAPeze kit; telomerase activity was not detected in the vector alone-transfected cells, but all of the cells transfected with the hTERT gene exhibited significant telomerase activity		n/d
[36]	n/d		protein WB, ICC	telomerase activity was detected by TRAP ladder on gel in BJ fibroblasts transfected with hTERT and rapidly proliferating HT1080 fibrosarcoma		n/d

*—no data (the authors did not perform the analysis); **—no exact data.

**Table 3:** Expression of specific markers in hTERT-immortalized cells.

Reference	Cell Type	Specific Cell Markers and Other Characteristics
[12]	normal and RDEB fibroblasts and keratinocytes	ICC expression of the respective specific markers: COLI, COLIV, FN, S100A4, and COLVII for fibroblasts; cytokeratin 14, loricrin, plectin, and C7 for keratinocytes
[37]	periodontal ligament fibroblasts (hPLF)	hPLFs and hPLF-hTERT positive for vimentin
[31]	hepatocyte-like hybrid cells	ICC positive expression of albumin, nuclear HNF4, the ASGP receptor, cytochrome P450 isotypes 1A2 and 3A4, glucuronosyltransferase isoforms UGT1A1 and UGT2B7, and Adipo Red for triglycerides; RNA isolates analyzed for the expression of alpha fetoprotein (AFP), α-1-antitrypsin (AAT), transthyretin (TTR), cytochrome P450 1A2 (CYP 1A2), cytochrome P450 3A4 (CYP 3A4), cytochrome P450 2C9 (CYP 2C9), hepatocyte nuclear factor 1 alpha (HNF1A), hepatocyte growth factor (HGF), and albumin (ALB)
[35]	AE pre-leukemia cell (fusion protein AML1- ETO)	expression of hTERT did not alter the multi-lineage competence of AE cells; no significant difference in the percentage of erythroid cells between AE-hTERT and AE-pBabe (measured by CD235a staining) in the methylcellulose colony assay; the erythroid potential of AE-hTERT cells was not changed; AE-hTERT cultures were able to generate lymphoid cells expressing CD19 and CD10 when cultured on MS-5 stroma with lymphoid cytokine supplementation
[33]	normal and scleroderma (SSc) fibroblasts	elevated expression levels of collagen I, connective tissue growth factor, and thrombospondin 1 mRNA; expression of other genes was not significantly changed; mRNA levels of nine profibrotic genes including COL1A1, TSP1, CCN2, Smad1, PLOD2, fibrillin 2 (FBN), collagen XI, desmin, and tenascin C (by qRT-PCR)
[34]	human mesenchymal stem cells (hMSCs)	i-hTERT hMSCs retain multipotentiality at PD 50 comparable to wild-type hMSCs and are able to undergo adipogenesis, osteogenesis, and chondrogenesis
[32]	human corneal epithelial cells (HCE)	cornea-specific keratin K3; ZO-1 expression of the normal human cornea was localized to the cell boundaries in a pattern consistent with the formation of typical tight junction complexes

**Table 4:** hTERT expression in hTERT-immortalized cells.

Reference	Cell Type	mRNA	Protein WB	ICC	Time of Assessment
[12]	fibroblasts and keratinocytes	n/d *	intense staining in all immortalized cell lines; almost no staining in primary cells	only nuclei are stained in the minority of cells (about 10%)	n/ed **
[36]	BJ foreskin fibroblasts	n/d	positive staining in hTERT cells, weaker than in fibrosarcoma HT1080 cells; no results in control cells for comparison	dot-like staining with DAB mainly in the nucleus; high in BJ + hTERT cells and fibrosarcoma HT1080 cells; absent in control cell	n/ed
[37]	human periodontal ligament fibroblasts (hPLFs)	no change	n/d	dot-like staining mainly in the cytoplasm, weaker in the few nuclei; increases in hTERT cells	about 20 passages
[34]	human mesenchymal stem cells (hMSCs)	very high expression in hTERT MSCS by RT PCR; weak expression in control cell lines	n/d	n/d	26 PDs vs. 9 PDs in wild-type cells
[13]	Hutchinson– Gilford progeria syndrome (HGPS) fibroblasts and healthy control fibroblasts	high levels induced shortly after the infection, no correlation of growth rate with mRNA; expression reduced at higher PDs (though cells continued growing at high proliferation rates)	n/d	n/d	three PD periods: low (between 7 and 20 PDs), medium (between 20 and 30 PDs), and high (above 47 PDs)
[29]	foreskin fibroblasts	high by qRT-PCR, very low in parental fibroblasts	high; no information about the level of parental fibroblasts	n/d	n/ed
[31]	hepatocyte-like hybrid cells	n/d	n/d	high expression in the cytoplasm and nuclei of 100% cells	n/ed

*—no data (the authors did not perform the analysis); **—no exact data.
